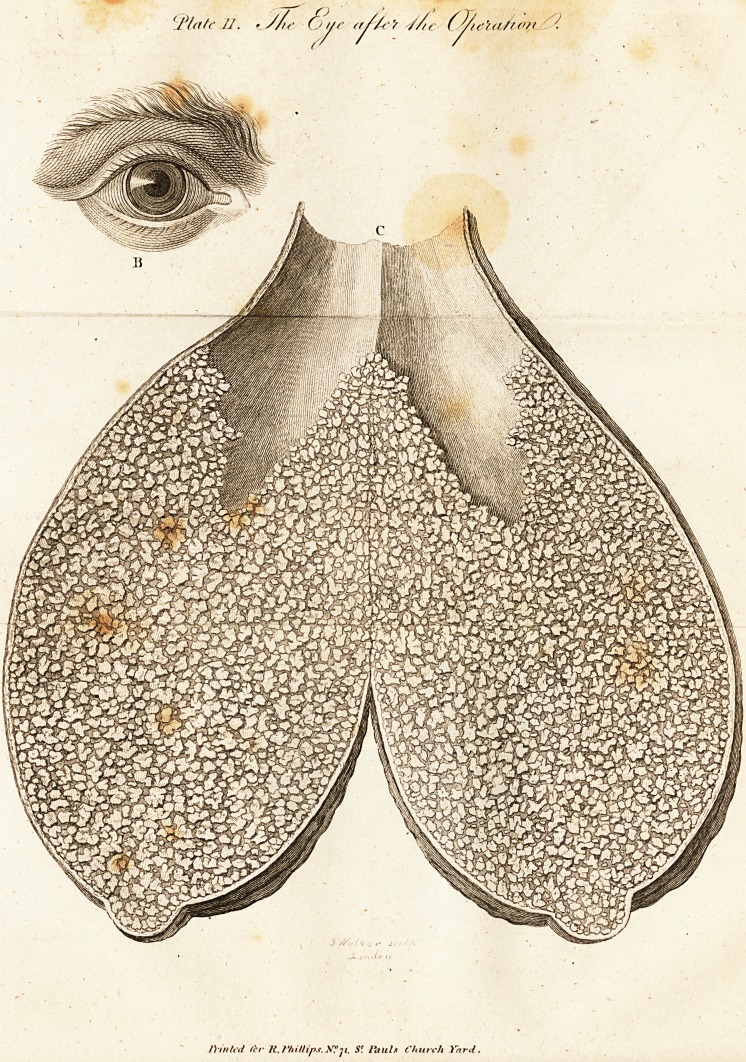# An Account of an Extraordinary Tumor on the Eye, Extirpated by F. Bouttatz, of Moscow, M. D. Fellow of the Medical Society of London, and Corresponding Member of the Royal Medical Society of Gottingen

**Published:** 1801-10

**Authors:** F. Bouttatz

**Affiliations:** Charlotte Street, Fitzroy Square


*Printed for R. Phillips, N°.71, S^t. Paul's Church Yard.*

*Plate II. The Eye after the Operation.*

J. Walker sculp.
London

Printed for R. Phillips, N.º 71, St. Pauls Church Yard.

THE

Medical and Phyfical Journal.

VOL. VI.] OCTOBER, 1801. [NO. XXXII.

*An Account of an extraordinary Tumor on the Eye, extirpated by* F. BOUTTATZ, *of Moscow, M. D. Fellow of the Medical Society of London, and corresponding Member of the Royal Medical Society of Gottingen.*

[ With Plates. ]

To the EDITORS of the MEDICAL and PHYSICAL JOURNAL.

Gentlemen,

YOUR valuable Journal being read by every fcientific practitioner, I beg you will do me the favour to infert the following cafe in your next Number.

*Charlotte Street, Fitzroy Square,*
*Sept.* 10, 1801.

I am, &c.

F. BOUTTATZ.

DURING my refidence in Switzerland, in 1797, I frequently met a man, from whofe left eye an uncommon excrefcence depended; but whenever I met him, I was prevented from more minutely examining him, as the company in which I was fhuddered at the fight of fuch a monftrous excrefcence, which, indeed, had a moft dreadful appearance. Being, however, impatient to inquire into the cafe of this man, I appointed him to meet me at Morge, where I frequently ftaid a confiderable time, and where I examined his eye, and found it in the following ftate. The excrefcence, (Tab. I.) depended from the left eye, in the form of a bag; its fhape refembled that of a pear, and had at the lower extremity a fmall protuberance. This bag was 7 ½ inches long, and 3 ½ inches in circumference. The point of this bag was hard, but it gradually grew fofter as it approximated the eye. Within the diftance of about an inch from the eye, it felt hollow, which made me hope that the bag did not take its origin from the furface of the cornea. This excrefcence could be eafily moved in any direction, and its colour exactly agreed with the complexion of the face, which was of a tawny hue, membranous, and wrinkled towards the point, as if folded. The man generally made uſe of a cloth to ſupport the excreſcence, the weight of which, as he told me, ſeemed to be drawing the eye out of the ſocket. He was forty-five years old, and had been afflicted fifteen years with this tumor, which he ſuppoſed was cauſed by an inflammation in the eye. This inflammation he reported to have been of very long duration, though not violent, and when it ſubſided a ſkin began to form itſelf upon the eye, and gradually increaſing, by degrees expanded itſelf, without being attended with intenſe pain. He felt, however, an obtuſe pain in the head, and in the ſocket of his eye, accompanied with a ſenſation as if ſomething was drawing it out of the orbit. The eye-lids were unnaturally diſtended, appearing to be larger than uſual. He aſſured me that he could diſtinguiſh day from night with this eye, notwithſtanding the bag that encloſed it, and he even could perceive when a lighted candle was brought into the room. This information made me conclude that the bag did not cover the whole ſuperficies of the pupil, which very probably was perfectly ſound. On examining the bag, I found that towards the interior corner its ſubſtance ſeemed to be thinner than in the lower parts; and upon putting my thumb upon that part, by means of which the man could diſtinguiſh light from darkneſs, he could not ſee. This induced me to think that the man might be delivered from his ſingular burden by means of an operation. I intimated my opinion to him, but he abſolutely refuſed to ſubmit to it. I offered him money, if he would only permit me to make an inciſion in that part of the bag through which he could diſtinguiſh light from darkneſs, in order to aſcertain whether his eye was injured or not; but all that I urged was to no effect. He left me, and I gave up all hopes of ever ſeeing him again.

I was deſirous of performing the operation for the following reaſons:

In the firſt place, as the bag was neither large in circumference, nor very thick near the pupil of the eye.

2. As there was no reaſon to apprehend that I ſhould meet with any large veſſel to induce conſiderable hæmorrhage, which, moreover, ſhould it take place, would rather be beneficial by moderating the inflammation that would ſucceed the operation.

3. As the bag being moveable, I had no reaſon to think that in all points it adhered to the ſuperficies of the eye.

4. This appeared the more probable, the man being able to diſtinguiſh light from darkneſs.

5. As it was highly probable, that the pain which the man felt felt in the corner of the eye and in his head, proceeded from the weight of the excrefcence, which might occafion a caries, or other mifchief, in the focket of the eye.

6. As that part, whence the perfon could diftinguifh light from darknefs, could ferve me as a guide in my operation, and determine me to go through, or to difcontinue it. For in cafe I fhould have found the eye injured, or the affertion of the perfon erroneous, I might have fuffered the incifion again to clofe without proceeding further.

After the lapfe of eight days the man came to me again, informing me that he had made up his mind to fubmit to the operation, if I would make him a prefent of money.

Having maturely pondered the motives which had determined my refolution, I was confident of being completely juftified in attempting an operation. I was the more refolved not to let this opportunity efcape, as a young phyfician but rarely meets with fuch a cafe.

Before I commenced the operation, I tied a bandage over the found eye, and then made an incifion with a biftoury in the internal corner of the eye, where the perfon could diftinguifh light from darknefs, acrofs the excrefcence clofe to the eye. I had fcarcely penetrated a few lines, before I found that the excrefcence was hollow, and that there was a confiderable cavity between it and the eye. Having enlarged the incifion fo much that I could conveniently introduce a finger, and cleanfe the wound with a fponge, the perfon told me that he could fee a little; which confirmed me in my hope that the eye would be found uninjured. The further I proceeded in my operation, the more firmly was I convinced that the eye was perfectly found. In making the incifion, I took care to follow the fhape of the eye, and to lay it open as much as poffible.

As foon as I had performed a circular incifion, the bag was entirely detached from the eye. I left juft as much of the excrefcence as was required to heal the edges of the wound without injuring the eye.

The hæmorrhage was inconfiderable in proportion to the wound, but my patient, neverthelefs, fainted away for about five minutes. I availed myfelf of this opportunity to cleanfe the wound, and to examine the eye more minutely, when I found that the excrefcence had not been any thing elfe but an unnatural extenfion of the conjunctiva, which probably had feparated itfelf from the eye, and formed that bag.

It would be highly important to know what external remedies were applied in the beginning of the inflammation, and during the progrefs of the difeafe; but this information one cannot expect to obtain from an ignorant patient, efpecially

 when when the difeafe is of many years ftanding, and many particulars neceffary to be known have efcaped the memory.

My patient foon recovered from his fwoon. In order to prevent a violent inflammation, as he was of a fanguine temperament, I ordered him to lofe ten ounces of blood, and apply cold lotions to the eye. I alfo kept the eye-lids open by means of fticking plaifter, to prevent them from irritating the wound, and occafioning a violent inflammation. The eye-lids, efpecially the lower one, were diftended in an unnatural manner.

I directed my patient to take ten grains of nitre every three hours, and to bathe his feet in warm water and muftard, in order to excite a counter ftimulus and affift in abating the inflammation of the eye. Towards evening the patient complained of violent pain in the cavity of his eye, and in his head. His pulfe was extremely ftrong and full, which induced me to bleed him a fecond time, and to draw about eight ounces of blood. Inftead of the cold lotions, I directed emollient cataplafms of mallows and camomile to be applied. His pain was very violent at night, and he could not fleep till towards morning. The next day the pain was lefs violent; the dreffing was not changed; the nitre was continued; and a common glyfter injected, in order to keep the body open.

The pain was inconfiderable on the third day. I opened the dreffing, when I found the edges of the wound inflamed, and very much fwelled. The eye was red and inflamed; the eye-lids were fwelled, and fomewhat inflamed. I ordered the emollient remedies to be continued; and as the patient was coftive, I prefcribed a purgative, which produced the defired effect. The fwelling had abated the next day, and the eye was lefs inflamed. The patient informed me that he had flept well. The diet was antiphlogiftic. As the edges of the wound began to fuppurate, and the pain had confiderably abated, the eye was dreffed twice a day; and as the fuppuration now grew more copious every day, and not the leaft fign of danger appeared, I caufed the edges of the wound to be moiftened at different times with a weak folution of extr. Saturn. in order to check its progrefs. The eye was wafhed with a compofition of opium and rofe-water, in order to cleanfe it from the matter which iffued upon it from the wound.

A few days after the folution of extr. Saturn. was increafed, I prefcribed gentle purgatives, and directed my patient to obferve the moft rigorous antiphlogiftic diet. The continuation of this treatment foon made the edges of the wound cicatrize; but the more they cicatrized, the more did the patient complain of pain in the eye, the caufe of which I could not at firft firſt diſcover; but when I examined the eye more cloſely, I found that the pain was occaſioned by the contraction of the wound producing a preſſure upon the eye, which determined me to raiſe the edge of the wound with a pair of forceps, and ſplit it with a biſtoury. The patient derived inſtant relief from this operation, and his pain left him entirely. The inciſion healed in a ſhort time, without any bad conſequences ariſing therefrom. The proud-fleſh, which appeared in different parts of the wound, being extirpated by means of cauſtics, the wound healed very faſt, and became as it were cartilaginous. Although the eye-lids were conſiderably diſtended, yet they did not entirely cover the edge of the excreſcence.

When I had performed the operation, I opened the bag (Pl. 2.) lengthways, in order to examine it. It was filled with a lardaceous ſubſtance from the point up to within an inch of the eye, and very much reſembled a ſteatoma, from which it however differed by the appearance of the fatty ſubſtance, which was not ſmooth like lard, but granulated and uneven. The point of the excreſcence had a protuberance which is well repreſented in the plate. The part where it adhered to the eye was hollow. The ſurface of the excreſcence was membranous, and contracted in wrinkles, weighing two pounds and two ounces. In form it reſembled a hydrocele.

---

### Explanation of the Plates.

Plate I. The appearance of the eye and tumor previous to the operation.

Plate II. 1. The eye after the operation. 2. Section of the ſac.

## Figures and Tables

**Plate I. f1:**
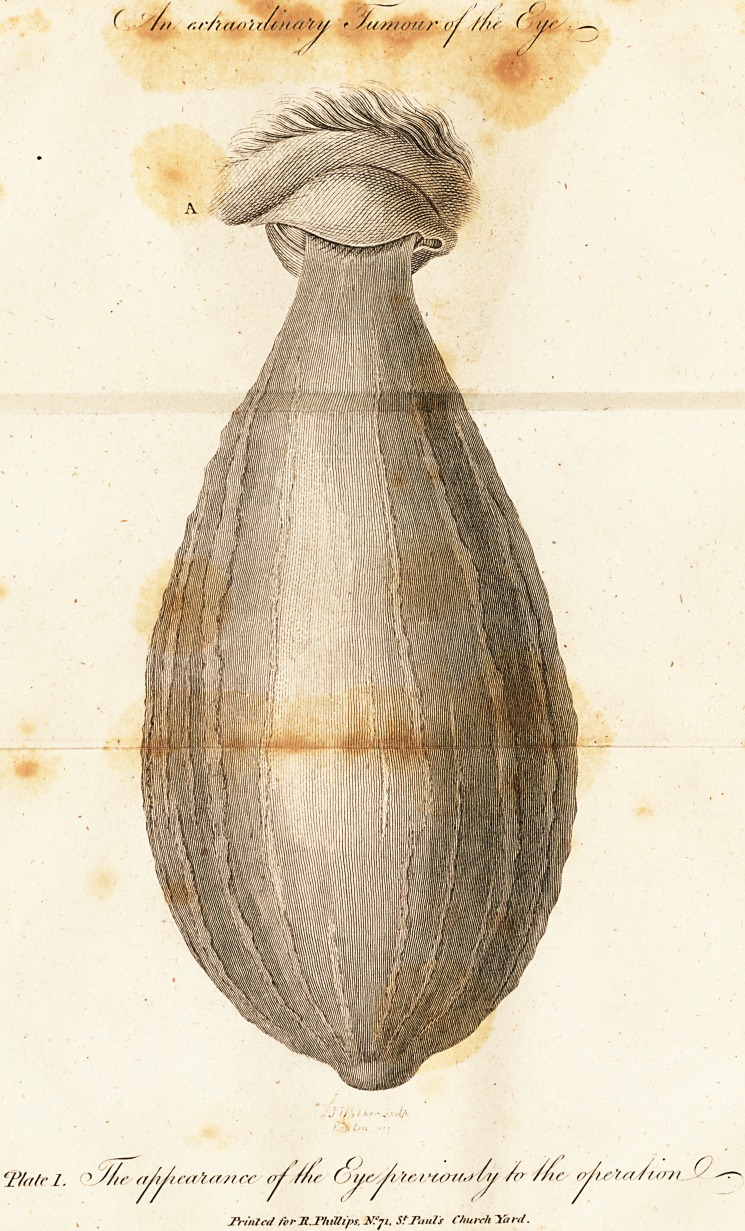


**Plate II. f2:**